# Carnitine Palmitoyltransferase System: A New Target for Anti-Inflammatory and Anticancer Therapy?

**DOI:** 10.3389/fphar.2021.760581

**Published:** 2021-10-26

**Authors:** Muyun Wang, Kun Wang, Ximing Liao, Haiyang Hu, Liangzhi Chen, Linlin Meng, Wei Gao, Qiang Li

**Affiliations:** ^1^ Department of Pulmonary and Critical Care Medicine, Shanghai East Hospital, Tongji University School of Medicine, Shanghai, China; ^2^ Department of Vascular Surgery, Shanghai Jiao Tong University Affiliated Sixth People’s Hospital, Shanghai, China; ^3^ Department of Traditional Chinese Medicine, Shandong University of Traditional Chinese Medicine, Jinan, China

**Keywords:** carnitine palmitoyltransferase (CPT), fatty acid oxidation (FAO), inflammatory diseases, cancers, CPT activator, CPT inhibitor

## Abstract

Lipid metabolism involves multiple biological processes. As one of the most important lipid metabolic pathways, fatty acid oxidation (FAO) and its key rate-limiting enzyme, the carnitine palmitoyltransferase (CPT) system, regulate host immune responses and thus are of great clinical significance. The effect of the CPT system on different tissues or organs is complex: the deficiency or over-activation of CPT disrupts the immune homeostasis by causing energy metabolism disorder and inflammatory oxidative damage and therefore contributes to the development of various acute and chronic inflammatory disorders and cancer. Accordingly, agonists or antagonists targeting the CPT system may become novel approaches for the treatment of diseases. In this review, we first briefly describe the structure, distribution, and physiological action of the CPT system. We then summarize the pathophysiological role of the CPT system in chronic obstructive pulmonary disease, bronchial asthma, acute lung injury, chronic granulomatous disease, nonalcoholic fatty liver disease, hepatic ischemia–reperfusion injury, kidney fibrosis, acute kidney injury, cardiovascular disorders, and cancer. We are also concerned with the current knowledge in either preclinical or clinical studies of various CPT activators/inhibitors for the management of diseases. These compounds range from traditional Chinese medicines to novel nanodevices. Although great efforts have been made in studying the different kinds of CPT agonists/antagonists, only a few pharmaceuticals have been applied for clinical uses. Nevertheless, research on CPT activation or inhibition highlights the pharmacological modulation of CPT-dependent FAO, especially on different CPT isoforms, as a promising anti-inflammatory/antitumor therapeutic strategy for numerous disorders.

## 1 Introduction

Lipids, which generally consist of triglycerides, cholesterol, phospholipids, and glycolipids, are hydrophobic molecules that have three basic functions, namely, energy storage, signal transduction, and membrane building. Initially considered as the reserves of static metabolic energy, these are now also considered as important components of various cellular signal transduction pathways. The roles of lipids in modulating host immune response, either in promoting or eliminating inflammation, have been of major clinical interest (Chen et al., 2019). Recently, lipid metabolism has been proved to be associated with various diseases, including acute and chronic inflammatory disorders and cancer. As one of the most important lipid substances *in vivo*, fatty acid (FA) utilization by β-oxidation is a major bioenergetic pathway that could be upregulated with prolonged fasting, exercise, or metabolic stress. FA oxidation (FAO) mainly occurs in the mitochondria and involves a series of reactions that result in the conversion of FA to acetyl-coenzyme A (acetyl-CoA). In the early 20th century, Franz (1904) elucidated the mechanisms underlying FA degradation by successive cyclic removal of two carbon units at a time, which subsequently initiated further studies on FAO (Schlaepfer and Joshi, 2020).

Compared with the transmembrane movement of short-chain and medium-chain FAs, the transport of long-chain FA is more difficult, thus becoming a key step of FAO. In the mid-1950s, Fritz (1955) determined the essential function of carnitine in the oxidation of long-chain FA in mammalian tissues. Subsequent studies by Bremer (1963) and Fritz and Yue (1963) led to a conceptual framework depicting how carnitine enables long-chain FA esterification to CoA in the extramitochondrial compartment to generate enzymes of β-oxidation in the mitochondrial matrix, thus circumventing the permeability issue of the inner membrane to acyl-CoA esters. Generally, the transfer of long-chain FAs into the mitochondria for oxidation occurs in a well-organized and regulated manner. Enzymes that facilitate this transfer are known as L-carnitine acetyltransferases; these catalyze the reversible transfer of acyl groups between L-carnitine and coenzyme A (CoA), resulting in the conversion of acyl-CoA esters into acyl-carnitine esters and vice versa (Schlaepfer and Joshi, 2020). Due to the impermeability of the mitochondrial inner membrane to long-chain CoA FA, this step in CoA and carnitine exchange is essential (McGarry and Brown, 1997). Among the enzymes, carnitine palmitoyltransferase (CPT) plays a rate-limiting role in FAO and thus has been recognized as a pivotal component of cellular metabolic homeostasis. CPT occurs in two isoforms, namely, CPT1 and CPT2, which are localized mainly in the mitochondria (Brosnan et al., 1973; McGarry and Brown, 1997) and play a crucial role in preserving their structural and functional integrity. In addition, CPT also facilitates adaptation to the environment, under both healthy and disease conditions (Roe, 2002). Therefore, intensive studies on CPT may help to understand in depth the pathogenesis of various diseases and explore a promising class of therapeutics.

## 2 Structure, Distribution, and Physiological Action of the Carnitine Palmitoyltransferase System

CPT1, CPT2, and carnitine-acylcarnitine translocase (CACT) play vital roles in the transport system for FA esterification in the mitochondrial membrane. The transmembrane protein CPT1 is located at the outer mitochondrial membrane, while CPT2 is in the inner of the mitochondrial membrane (Fraser et al., 1997). Unlike the unique form of CPT2 (Demaugre et al., 1990), three tissue-specific isoforms of CPT1 have been identified: the liver isoform (L-CPT-1, CPT1A), muscle isoform (M-CPT-1, CPT1B), and brain isoform (B-CPT-1, CPT1C) (Britton et al., 1995; Yamazaki et al., 1996; Price et al., 2002). CPT1A, with its full-length cDNA clone isolated from rat liver that predicted a protein of 773 amino acids (Esser et al., 1993), is characterized by tight mitochondrial membrane binding, which would lose activity once removed from the membrane. Compared with CPT1A, CPT1B consists of 772 amino acids (Cox et al., 1998; van der Leij et al., 2000) and has lower affinity for substrate carnitine (McGarry and Brown, 1997). A study has demonstrated that homozygous deletions in CPT1B are lethal in mouse (Ji et al., 2008). The protein primary sequence of CPT1C is larger (798 amino acids) than the two other isoforms. Although CPT1C tends to adopt the same membrane topology as CPT1A, its enzyme activity is extremely low or undetectable (Hada et al., 2014). First identified in 1990 (Woeltje et al., 1990a; Woeltje et al., 1990b), the cDNA sequence of CPT2 predicted a nascent product of 658 amino acids in both rats and humans. Unlike CPT1, CPT2 does not contain a single polypeptide with both the inhibitor binding and catalytic domains (Bonnefont et al., 2004).

CPT1A is the primary isoform and is found in the liver, spleen, kidneys, lungs, intestines, pancreas, brain, and ovaries (Brown et al., 1997; McGarry and Brown, 1997). CPT1B is predominant in the skeletal muscle, adipose tissue, heart, and testis (Esser et al., 1996), whereas CPT1C is mainly expressed in the brain and is downregulated in the testis, ovaries, small intestine, and colon (Price et al., 2002). Microcosmically, CPT1A and CPT1B are both located in the outer membrane of the mitochondria, whereas CPT1C is localized to both the endoplasmic reticulum and mitochondria (Dai et al., 2007; Sierra et al., 2008). CPT1 isoform switching in the mitochondria has been established during the development of rat heart; although CPT1A represents a minor constituent of the CPT complex in the adult rat heart, its contribution is much greater in newborn animals (Brown et al., 1995)*.* CPT2 is a ubiquitous enzyme in rats and humans (Demaugre et al., 1990; Woeltje et al., 1990a; Woeltje et al., 1990b)*.*

**FIGURE 1:**
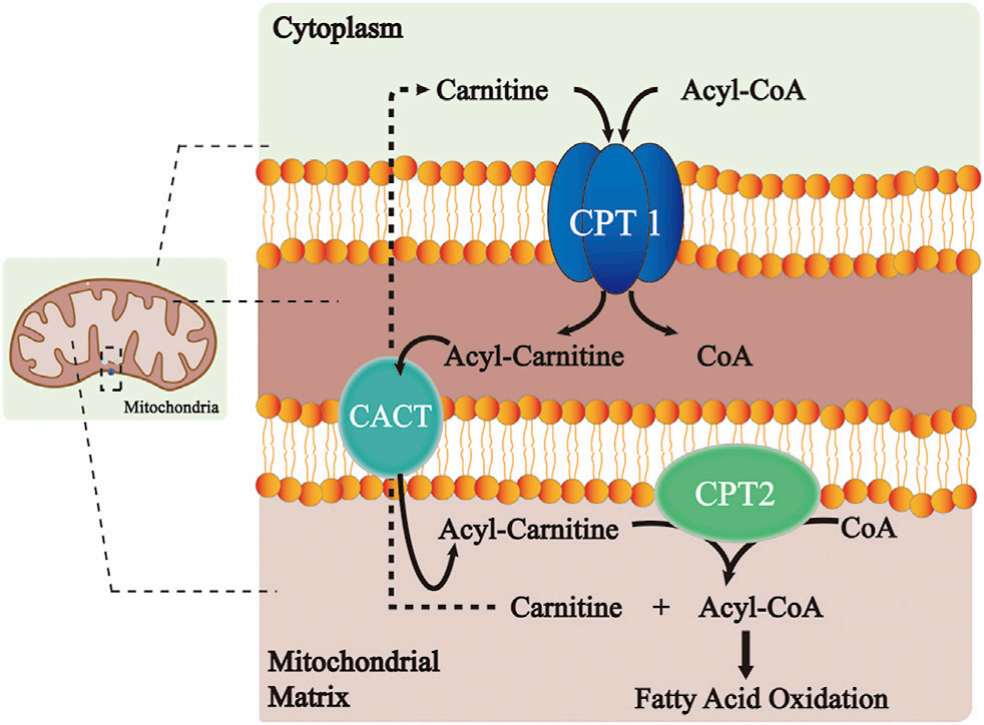
Role of the CPT system in the long-chain FA oxidation. CPT1 at the outer mitochondrial membrane catalyzes the conversion of long-chain acyl-CoA along with carnitine to long-chain acylcarnitine and CoA. The transesterified acylcarnitines are then transferred from cytosol into intermembrane space and the remaining acyl of acylcarnitine is changed back to CoA on the inner membrane catalyzed by CPT2, which is next available for β-oxidation. Meanwhile, the released carnitine is returned to the intermembrane space of the mitochondrion through the CACT and is available for the re-transport of FA. Abbreviations: CPT, carnitine palmitoyltransferase; CACT, carnitine–acylcarnitine–translocase; and CoA, coenzyme A.

The CPT system is an important intermediate of lipogenesis and a vital mechanism for the homeostasis of FA metabolism (Figure 1). CPT1A and CPT1B at the outer mitochondrial membrane catalyze the first transport step of lipid metabolism, in which the long-chain acyl-CoA and carnitine are converted into long-chain acylcarnitine and CoA. The transesterified acylcarnitines are then transferred from the cytosol into the intermembrane space (Eaton et al., 1996; Console et al., 2014) and the remaining acyl of acylcarnitine is converted back to CoA on the inner membrane and catalyzed by CPT2, which is then available for β-oxidation (Joshi and Zierz, 2020). Meanwhile, the released carnitine signal transduction is returned back to the intermembrane space of the mitochondrion through the CACT and available for the re-transport of FA (Joshi and Zierz, 2020). Comparatively, CPT1C does not serve a key role in FAO. However, it shows significant effects on neuronal oxidative metabolism, energy homeostasis, and cell senescence (Lee and Wolfgang, 2012; Reilly and Mak, 2012; Guan et al., 2019). In terms of the underlying molecular pathway, the peroxisome proliferator-activated receptor (PPAR) family is a key transcription factor in the development of FAO. Studies have shown that PPAR activation controls the levels of intracellular free fatty acids (FFAs) (Castaño et al., 2018; Ye et al., 2019). Furthermore, the expression and activity of the CPT system increase with PPAR activation, thus manipulating FA metabolism.

## 3 Implications of Carnitine Palmitoyltransferase System in Inflammatory Diseases and Cancers

**FIGURE 2:**
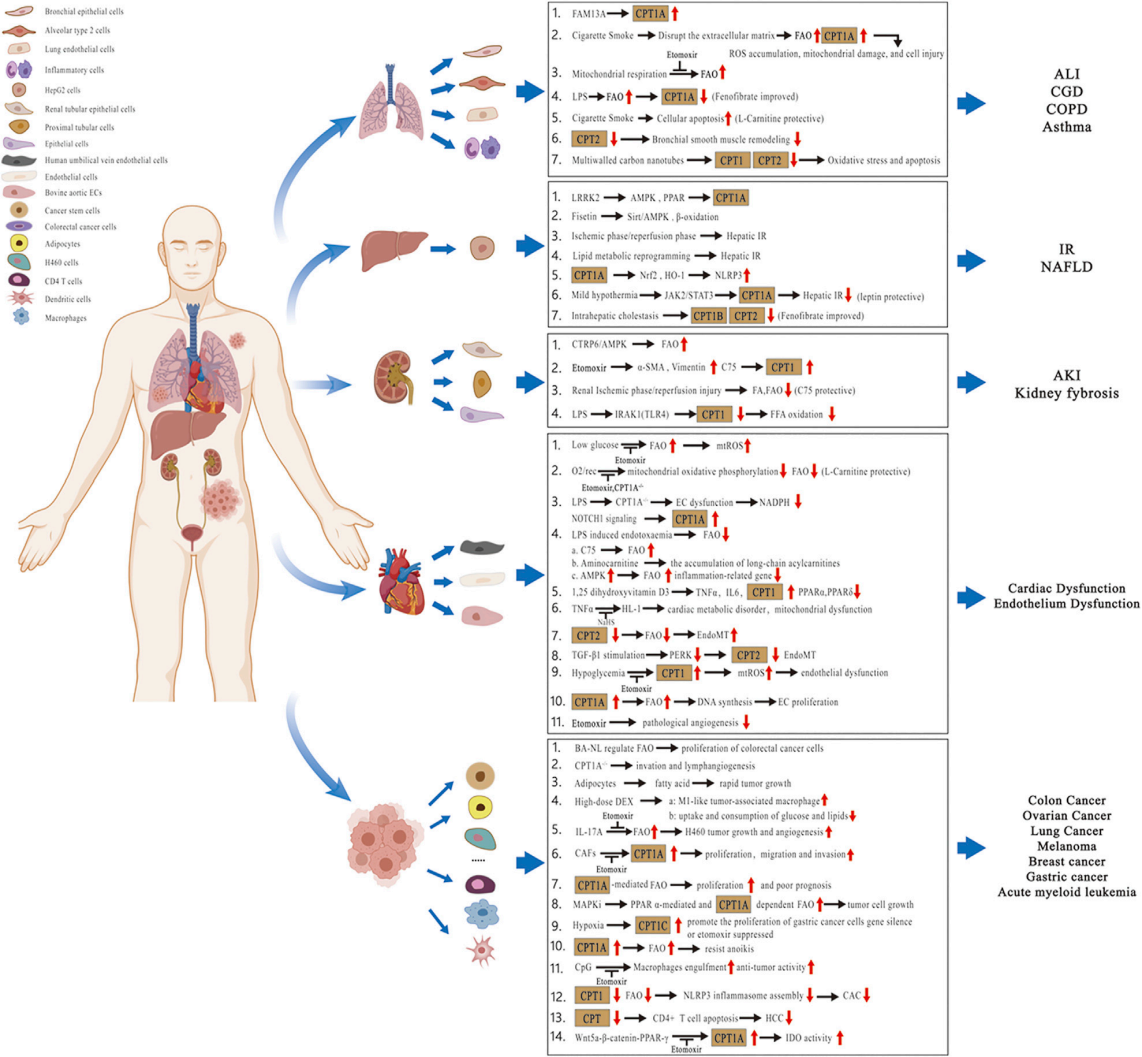
Pathophysiological role of the CPT system in different tissues or organs. Related diseases include COPD, asthma, ALI, CGD, NAFLD, hepatic IR injury, kidney fibrosis, AKI, cardiovascular disorders, and cancer. Abbreviations: COPD, chronic obstructive pulmonary disease; ALI, acute lung injury; CGD, chronic granulomatous disease; NAFLD, nonalcoholic fatty liver disease; IR, ischemia–reperfusion; AKI, acute kidney injury; CPT, carnitine palmitoyltransferase; FA, fatty acid; FFA, free fatty acid; FAO, fatty acid oxidation; FAM13A, family with sequence similarity 13 member A; ROS, reactive oxygen species; mtROS, mitochondrial ROS; LPS, lipopolysaccharide; LRRK2, leucine-rich repeat kinase 2; AMPK, AMP-activated protein kinase; Sirt, sirtuin; PPAR, peroxisome proliferator-activated receptor; Nrf2, nuclear factor erythroid-2–related factor 2; HO-1, heme oxygenase-1; NLRP3, nucleotide-binding oligomerization domain-like receptor protein 3; JAK2/STAT3, Janus kinase 2/Signal transducer and activator of transcription 3; CTRP6, C1q/tumor necrosis factor‐related protein 6; α-SMA, α-smooth muscle actin; IRAK1, interleukin-1 receptor–associated kinase 1; TLR4, toll-like receptor 4; O_2_/rec, hyperoxia followed by air recovery; ECs, endothelial cells; TNFα, tumor necrosis factor-α; IL6, interleukin 6; NaHS, sodium hydrosulfide; EndoMT, endothelial-to-mesenchymal transition; TGF-β1, transforming growth factor-β1; PERK, protein kinase R-like endoplasmic reticulum kinase; BA-NL, betulinic acid–loaded nanoliposomes; DEX, dexamethasone; IL-17A, interleukin 17A; CAFs, cancer-associated fibroblasts; MAPKis, mitogen-activated protein kinase inhibitors; CAC, colitis-associated cancer; HCC, hepatocellular carcinoma; and IDO, indoleamine 2,3-dioxgenase-1.

**FIGURE 3:**
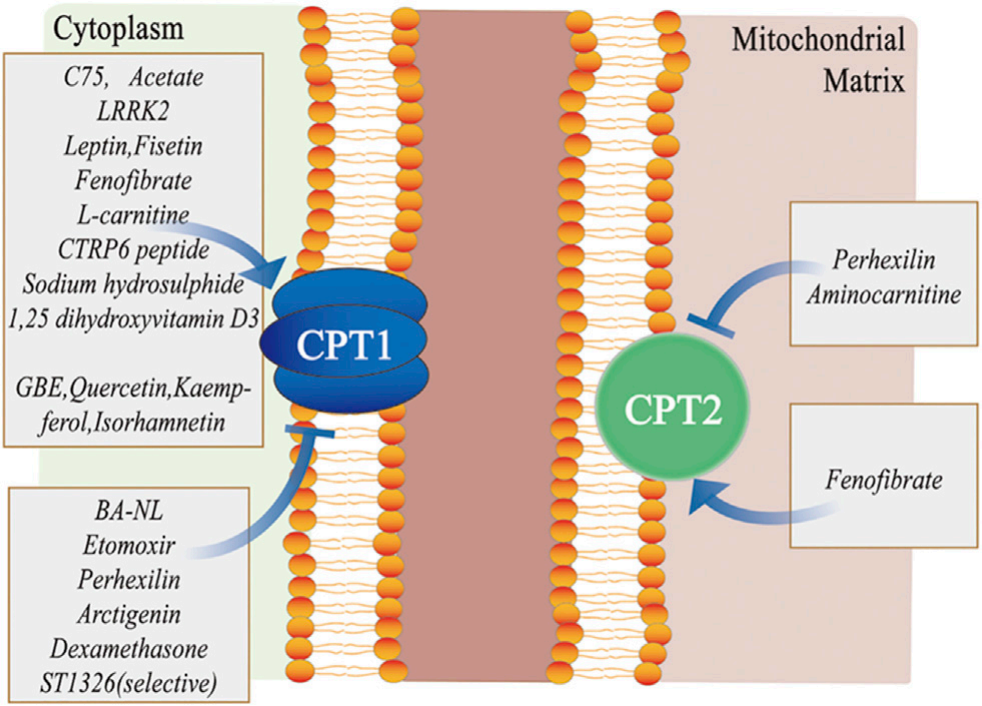
Different pharmacological modulators targeting the CPT system. C75, acetate, LRRK2, leptin, fisetin, fenofibrate, L-carnitine, CTRP6 peptide, sodium hydrosulphide, 1, 25-dihydroxyvitamin D3, GBE, quercetin, kaempferol, and isorhamnetin primarily activate CPT1, whereas BA-NL, etomoxir, perhexilin, dexamethasone and ST1326 mainly inhibit CPT1. Similarly, fenofibrate activates CPT2, while perhexilin and aminocarnitine inhibit CPT2. Abbreviations: LRRK2, leucine-rich repeat kinase 2; CTRP6, C1q/tumor necrosis factor–related protein 6; GBE, ginkgo biloba extract; CPT, carnitine palmitoyltransferase; and BA-NLs, betulinic acid–loaded nanoliposomes.

In recent years, studies have focused on the contribution of lipid metabolic pathways on the pathogenesis of multiple disorders. Considering the modulatory effects and clinical implications of lipid molecules in different tissues or organs, we summarize the pathophysiological role of the CPT system in many diseases of acute and chronic inflammation as well as cancer in this review. These diseases include chronic obstructive pulmonary disease (COPD), bronchial asthma, acute lung injury (ALI), chronic granulomatous disease (CGD), nonalcoholic fatty liver disease (NAFLD), hepatic ischemia–reperfusion (IR) injury, kidney fibrosis, acute kidney injury (AKI), cardiovascular disorders, and cancer (Figure 2; Table 1). We also concentrate on the current knowledge on pharmacological modulators targeting the CPT system from preclinical evaluation to clinical trials in managing these diseases (Figure 3; Table 2).

**TABLE 1:** The role of CPT in inflammatory disease.

Targets	Associated disease	Types of CPT	Major outcome(s)	References
Liver	Liver injury	CPT1B and CPT2	Decreased in intrahepatic cholestasis model	Zhao et al. (2017)
		CPT1A	Promoted oxidative stress	Luo et al. (2021)
		CPT1A	Decreased in HepG2 cells; inhibited inflammation	Wei et al. (2014)
		CPT1A	Suppressed inflammation	Lin et al. (2020)
		CPT1A	JAK2/STAT3-CPT1A–dependent FAO attenuated injury	Wang W. et al. (2020)
		CPT1 and CPT2	L-carnitine elevated its transcription and activity	Karlic et al. (2002)
		CPT	Carnitine ingestion during pregnancy increased liver CPT activity and fetal carnitine concentrations	Xi et al. (2008)
	NAFLD	CPT1	Improved the symptoms of the disease	Liou et al. (2018)
		CPT1	Impaired CPT1 induced hepatic dysfunction and inflammation	Schröder et al. (2016)
	HCC	CPT	Upregulated CPT elevated apoptosis of CD4^+^ T cells and promoted HCC formation in NAFLD	Brown et al. (2018)
Cardiovascular System	Cardiac dysfunction	CPT1	Downregulated CPT1 induced myocardial dysfunction	Eaton et al. (2003)
		CPT1	Decreased in heart induced by LPS	Fukumoto et al. (2002)
		CPT1	Upregulated in diabetic rats	Lee et al. (2014)
		CPT1	Downregulated CPT1 induced the injury	Lee et al. (2019)
		CPT1 and CPT2	Downregulated CPT induced cardiac injury during endotoxemia	Makrecka-Kuka et al. (2020)
	Endothelium Dysfunction	CPT1A	Loss of CPT1A elevated oxidative stress and promoted endothelial barrier disruption	Kalucka et al. (2018)
		CPT1	Downregulated CPT1 increased atherosclerosis	Fruchart et al. (1999)
		CPT2	Genetic disruption potentiated EndoMT	Xiong J. et al. (2018)
Pulmonary	Asthma	CPT1	Raised in asthmatic mice	Al-Khami et al. (2017)
		CPT2	Increased in asthmatic bronchial SMC	Esteves et al. (2021)
	COPD	CPT1A	CS increased CPT1A and FAO	Jiang et al. (2017)
		CPT1	CS increased CPT1 expression and promote FAO	Agarwal et al. (2014)
	ALI	CPT1B	Decreased CPT1B increased mortality; increased expression and decreased activity in aged ALI mice	Gibbs et al. (2021)
		CPT1A	CPT1A inhibition or depletion aggravated EC apoptosis and lung injury	Yao et al. (2019)
Kidney	Diabetic nephropathy	CPT	Aggravated mitochondrial ROS accumulation in kidney cortical tubules	Rosca et al. (2012)
	Kidney fibrosis	CPT1A	Decreased during the disease	Xie et al. (2021)
		CPT1A	Overexpression of CPT1A showed protective effects	Miguel et al. (2021)
	IR injury	CPT1	Upregulated CPT1 improved renal function	Idrovo et al. (2012)
Colon	Colorectal cancer	CPT1A	Exposure to adipocytes or FA upregulated CPT1A	Xiong et al. (2020)
		CPT1A	CPT1A activation induced anoikis-resist	Wang et al. (2018)
		CPT1A	Low expression in primary tumor tissues while high expression in CAFs	Peng et al. (2021)
	CAC	CPT1	Suppressed CPT1 inhibited NLRP3 assembly in macrophages	Qiao et al. (2020)
Breast	Breast cancer	CPT1A	CPT1A^−/−^ abolished invasion and lymphangiogenesis	Xiong Y. et al. (2018)
		CPT1A	Upregulated in patients; a new biomarker for the diagnosis	Tan et al. (2021)
		CPT1A	Increased in doxorubicin-treated tumours *in vivo*	Petővári et al. (2020)
		CPT1C	Conferred rapamycin resistance on breast cancer cells	Reilly and Mak (2012)
Blood	AML	CPT1A	Overexpression predicted poor clinical outcome	Mao et al. (2021)
	Lymphoblastic leukemia	CPT1 and CPT2	Highly expressed in chronic lymphoblastic leukemia cells	Gugiatti et al. (2018)
	Leukemia	CPT	Downregulated CPT led to death of the leukemic cells	Liu et al. (2016)
Pancreas	Pancreatic cancer	CPT1C	Downregulated CPT1C inducted tumor senescence	Guan et al. (2019)
	Pancreatic ductal adenocarcinoma	CPT1A	Strengthened the antitumor immunity of CpG-treated macrophages	Liu et al. (2019)
Skin	Melanoma	CPT1A	Inhibited CPT1A led to apoptosis in MAPKi-treated cells	Aloia et al. (2019)
			Increased the tumor-mediated immune tolerance	Zhao et al. (2018)
Muscle	Muscle dysfunction	CPT2	A conceptual overview on CPT2 deficiency	Joshi and Zierz (2020)
Nervous	Neuronal dysfunction	CPT1C	Played alternative role in neuronal oxidative metabolism	Lee and Wolfgang (2012)
Gastric	Gastric Cancer	CPT1C	Associated with poor prognosis; promoted proliferation of cancer cells	Chen et al. (2020)

Abbreviation: CPT, carnitine palmitoyltransferase; NAFLD, nonalcoholic fatty liver disease; HCC, hepatocellular carcinoma; JAK2/STAT3, Janus kinase 2/signal transducer and activator of transcription 3; EndoMT, endothelial-to-mesenchymal transition; SMC, smooth muscle cell; CS, cigarette smoke; ALI, acute lung injury; EC, endothelium cells; IR, ischemia–reperfusion; FA, fatty acid; CAC, colitis-associated-cancer; CAFs, cancer-associated fibroblasts; NLRP3, nucleotide-binding oligomerization domain-like receptor protein 3; AML, acute myeloid leukemia; and MAPKi, mitogen-activated protein kinase inhibitors.

**TABLE 2:** The development status of CPT inhibitors/activators.

Drugs	Utility	Preclinical/Clinical Study	Model	Dose	References
Etomoxir	CPT1 inhibitor	Preclinical	Murine model	50 mg/kg	Al-Khami et al. (2017)
		Preclinical	HUVECs, H460 cell line	40 μM	Wang et al. (2019)
		Preclinical	16HBE cell line	50 μM	Jiang et al. (2017)
		Preclinical	Patients' CAFs	50 μM	Peng et al. (2021)
		Preclinical	Murine model, HKC8 cell line	30 mg/kg in mice, 40 µM in cells	Kang et al. (2015)
		Preclinical	Human primary BSM cells	10 nM	Esteves et al. (2021)
		Preclinical	Murine model	5 mg/kg	Wang W. et al. (2020)
		Preclinical	MLE-12, HEK-293T cell line	100 µM	Cui et al. (2019)
		Preclinical	Murine model, human primary HUVEC and EC, mice primary EC	30 mg/kg in mice, 100 μM in cells	Kalucka et al. (2018)
		Preclinical	Mice primary BMDMs	200 μM	Liu et al. (2019)
		Preclinical	Murine model, THP-1 cell line	2 mg/kg in mice, 10 μM in cells	Qiao et al. (2020)
		Preclinical	Murine model, mice primary DCs	25 mg/kg/day in mice, 100 μM in cells	Zhao et al. (2018)
Perhexilin	CPT1 and 2 inhibitor	Preclinical	Human primary BSM cells	10 nM	Esteves et al. (2021)
		Preclinical	Murine model, human primary CLL cells	8 mg/kg in mice, 5–10 μM in cells	Liu et al. (2016)
		Preclinical	Murine model	8 mg/kg	Brown et al. (2018)
ST1326	CPT1A selective inhibitor	Preclinical	Primary AML cells, AML cell line	6 μM or 10 μM	Mao et al. (2021)
Dexamethasone	CPT1A inhibitor	Preclinical	Murine model, LLC cells	50 mg/kg in mice, 1 μM in cells	Xu et al. (2020)
Betulinic acid–loaded nanoliposomes	CPT1A inhibitor	Preclinical	HCT116 cell line	50, 100, or 200 µM	Wang G. et al. (2020)
Arctigenin	CPT1A inhibitor	Preclinical	Murine model, THP-1 cell line, mice primary BMDMs	25, 50 mg/kg in mice; 3, 10, and 30 μM in cells	Qiao et al. (2020)
L-carnitine	CPT activator	Clinical	Patients	1 g/day	Nemati et al. (2019)
				12 g one dose	Evans et al. (2019)
				750 mg/day	Malek et al. (2016)
				1,000 mg/d	Lee et al. (2015)
				12 g one dose	Puskarich et al. (2015)
				20 mg/kg	Savica et al. (2005)
				2 g/day	Borghi-Silva et al. (2006)
Fenofibrate	CPT1B and CPT2 activator	Preclinical	Murine model	200 mg/kg	Zhao et al. (2017)
	CPT1A activator	Preclinical	Murine model	100 mg/kg	Cui et al. (2019)
C75	CPT1 activator	Preclinical	Rat model	3 mg/kg	Idrovo et al. (2012)
		Preclinical	Murine model	15 mg/kg	Kang et al. (2015)
1,25 dihydroxyvitamin D3	CPT1 activator	Preclinical	Rat model	150 ng/kg	Lee et al. (2014)
Sodium hydrosulphide	CPT1 activator	Preclinical	HL-1 cell line	100 µM	Lee et al. (2019)
Fisetin	Increased CPT1, but not CPT2	Preclinical	Murine model, FL83B cell line	20 mg/kg in mice, 3–100 μM in cells	Liou et al. (2018)
Ginkgo biloba extract, quercetin kaempferol, and isorhamnetin	Up-regulate CPT1A	Preclinical	HepG2 cell line	200 μg/ml GBE, 20 μg/ml quercetin, 20 μg/ml kaempferol, or 8 μg/ml isorhamnetin	Wei et al. (2014)
Acetate	CPT1A activator	Preclinical	Murine model, human primary HUVEC and EC, mice primary EC	0.5 M in mice, 500 μM in cells	Kalucka et al. (2018)
CTRP6 peptide	CPT1A activator	Preclinical	HK-2 cell line	2 μg/ml	Xie et al. (2021)
Leucine-rich repeat kinase 2	CPT1A activator	Preclinical	HepG2 cell line	200 or 400 μM	Lin et al. (2020)
Leptin	CPT1 activator	Preclinical	Murine model	5 mg/kg	Wang W. et al. (2020)

Abbreviation: CPT, carnitine palmitoyltransferase; HUVECs, human umbilical vein endothelial cells; CAFs, cancer-associated fibroblasts; BSM, bronchial smooth muscle; EC, endothelium cells; BMDMs, bone marrow–derived macrophages; DCs, dendritic cells; CLL, chronic lymphocytic leukemia; AML, acute myeloid leukemia; LLC, Lewis lung carcinoma; and CTRP6, C1q/tumour necrosis factor–related protein 6.

### 3.1 Pulmonary Diseases

The lung is seldom considered as a metabolic organ. However, active lipid metabolism occurs in lung tissues, especially within the alveolar area, where surfactant homeostasis is exquisitely regulated to ensure continuous optimal function in each respiration cycle. Metabolic disturbance of the lipid profile induces excess inflammation, oxidative stress, and cellular apoptosis, which has been proven to be involved in the occurrence and development of various lung diseases.

#### 3.1.1 Chronic Obstructive Pulmonary Disease

COPD is a major worldwide health problem that is increasing in prevalence and mortality (Rennard and Drummond, 2015). The progressive lung condition is characterized by an irreversible airflow limitation associated with an abnormal inflammatory response in the airway and is mostly attributable to noxious particles or gases (Mizumura et al., 2018; Gong et al., 2019). Among these, cigarette smoke (CS) is the major risk factor for the development of COPD, which accounts for at least 75% of the deaths (Gong et al., 2019). Despite increasing epidemiologic evidence linking lipid metabolism to CS-induced emphysema (Lundström et al., 2011; Zehethofer et al., 2015), the regulatory effects of FAO and CPT on COPD pathogenesis remains unclear (Jiang et al., 2017).

CS exposure has been reported to promote FAO and mitochondrial respiration, along with an increased expression of CPT1 in the airway epithelial cells (EpiCs) (Agarwal et al., 2014; Jiang et al., 2017; Gong et al., 2019). Genome-wide association studies on COPD have demonstrated that FAM13A (family with sequence similarity 13 member A) enhances FAO by upregulating CPT1A expression, while chemical or genetic inhibition of FAO attenuates the accumulation of mitochondrial-derived reactive oxygen species (ROS) and cell death induced by CS exposure *in vitro* and *in vivo* (Jiang et al., 2017). Additionally, CS exposure also disrupts the extracellular matrix during COPD (Shapiro and Ingenito, 2005), which could subsequently promote FAO in EpiC (Schafer et al., 2009). In terms of the specific mechanism, a recent study suggested that the increased FAO and CPT expression by CS challenge in airway EpiC might exploit fat storage in adipose tissues to meet elevated FA demands within the lungs under stress conditions. By metabolic adaptation, the cells are able to generate ATPs to meet their energy needs. However, sustained elevation in FAO and CPT could disturb the metabolic homeostasis of cells and be harmful to their fate and functions. This viewpoint has been supported by observations of reduced mitochondrial ROS production and improved human bronchial EpiC viability with treatment using a CPT1A inhibitor, etomoxir, after CS exposure (Jiang et al., 2017).

However, enhanced FAO and CPT expression by L-carnitine has been proven to be beneficial to emphysema or COPD (Gong et al., 2019). L-carnitine, which is the critical metabolite in the transport of long-chain FAs into the mitochondria for subsequent β-oxidation, is downregulated in the lungs of mice with emphysema (Conlon et al., 2016). L-carnitine is the substrate for CPT1 and can increase its gene and protein expression, thus promoting FAO (Karlic et al., 2002; Xi et al., 2008). Gong et al. (2019) have reported that L-carnitine promotes CPT1A gene expression in lung EpiCs, which in turn imparts a protective effect on CS-induced cellular apoptosis. Furthermore, L-carnitine preserves FAO after CS challenge in lung EpiC, thus preventing lung injury and subsequent emphysema (Petrache et al., 2005; García-Lucio et al., 2018). In an animal emphysema model induced by elastase, L-carnitine also exhibited a significant protective effect (Conlon et al., 2016). In a clinical study, Borghi-Silva et al. (2006) recruited moderate-to-severe COPD patients and conducted oral L-carnitine supplementation for 6 weeks, which showed improved exercise tolerance and inspiratory muscle strength. The exact influence of the CPT system to COPD development and the specific mechanism require further investigation.

#### 3.1.2 Acute Lung Injury

ALI and its severe form, acute respiratory distress syndrome (ARDS), are common respiratory critical syndromes with no effective therapeutic intervention. They are triggered by a variety of direct or indirect pulmonary insults, and their complex pathophysiology is yet to be fully understood. One group of researchers showed that profound impairment in cellular oxygen consumption is one of the pathological hallmarks in the lungs of patients with the pathogen-induced ALI. In the murine model of lipopolysaccharide (LPS)–induced ALI, severely impaired FAO in alveolar EpiCs participated in the inflammatory response and lung injury, which might be attributed to the decreased expression of key mediators involved in FAO, such as CPT1A, and could partly be counteracted by treatment with a PPARα agonist, fenofibrate (Cui et al., 2019). Hyperoxia or positive pressure ventilation induces sustained lung injury in neonates, which is likely due to metabolic dysregulation in pulmonary endothelial cells (ECs). In a hyperoxia-exposed newborn murine model, pharmaceutical inhibition using etomoxir or genetic deletion of *CPT1A* aggravated EC apoptosis and lung injury, while treatment with L-carnitine attenuated the pathological changes (Yao et al., 2019). Elevated age is a risk factor for the poor outcomes of ALI/ARDS. Using an LPS-triggered ALI model in adult and aged mice, Gibbs et al. (2021) assessed age-related alterations in lung inflammation, muscle injury, and metabolism. They observed that etomoxir administration resulted in an increase in the mortality of aged but not adult ALI mice, thereby confirming that the CPT system is essential for survival from severe lung injury and indicating that adult mice have improved resilience to FAO inhibition. Furthermore, CPT1B in the skeletal muscles of aged ALI mice showed a distinct phenotype with its upregulated expression and decreased activity relative to adults, suggesting its correlation to the adverse age-related outcomes of ALI/ARDS.

#### 3.1.3 Bronchial Asthma

As another common chronic respiratory disease, the pathophysiology of bronchial asthma (or asthma) is orchestrated by various inflammatory cells and mediators in close communication with airway structural cells, including EpiCs and smooth muscle cells (SMCs). Increasing evidence has linked energy metabolism to the differentiation, function, and longevity of these inflammatory and structural cells. In allergen-induced murine models, Al-Khami et al. (2017) reported a significant increase in CPT1 expression in the bronchial epithelium and infiltrated inflammatory immune cells of asthma mice. Furthermore, the pharmacologic inhibition of CPT1 by etomoxir decreased airway hyperresponsiveness, inflammatory cell infiltration, and cytokine production associated with the disease. Similarly, Esteves et al. (2021) found a metabolic switch toward mitochondrial β-oxidation with an increased rate of mitochondrial respiration and a higher level of CPT2 in asthmatic bronchial SMC, whereas blocking CPT2 by either etomoxir or perhexiline drastically reduced the proliferation of asthmatic bronchial SMCs and remodeling in bronchial smooth muscles.

#### 3.1.4 Chronic Granulomatous Disease

CGD is a primary immunodeficiency syndrome that is characterized by defects in respiratory burst of phagocytes, leading to serious and life-threatening infections (Squire et al., 2020). Studies have suggested that disrupted lipid metabolism and suppressed mitochondrial FAO contribute to the pathophysiology of granulomatous lung disease (Huizar et al., 2013; Soliman et al., 2020). In a murine model of pulmonary granulomatous inflammation, PPARγ expression and activity in alveolar macrophage were significantly reduced 60 days after multi-walled carbon nanotube (MWCNT) exposure. In macrophage-specific PPARγ knock-out mice, granuloma formation was much more extensive than in the wild-type after MWCNT challenge (Soliman et al., 2020). With enhanced mitochondrial FAO and CPT expression mediated by PPARγ activation, Soliman et al. discovered that MWCNT instillation reduced the mRNA expression of CPT1, CPT2, and PPARγ coactivator 1 alpha in permeabilized bronchoalveolar lavage cells, accompanied by elevated oxidative stress in alveolar macrophages and inflammatory injury of murine lung tissues (Huizar et al., 2013).

According to the current evidence, CPT activation can exert either a beneficial or harmful effect during the development of pulmonary diseases. For example, CS exposure could either elevate or decrease FAO and CPT expression of EpiCs in distinct COPD models. This may be explained by the changing metabolic state of injured cells or organs during the pathological process of disease. Inhibition of CPT-dependent FAO impairs the energy metabolism of cells; meanwhile, the continuous activation of the CPT system may also contribute to mitochondrial dysfunction and excess ROS production, which further aggravate cellular damage. Accordingly, it is necessary to identify the basic metabolic profile of certain cells and the pathophysiological condition of the diseases before conducting the experiment.

### 3.2 Liver Diseases

Mitochondrial FAO is the primary pathway for FA metabolism and performs a key role for energy homeostasis in the liver (Li and Davie, 2010; Singh et al., 2012). Abnormal FAO and CPT1A expression have been shown to participate in the development of NAFLD and hepatic IR injury (Wei et al., 2014). The expression of FAO-relevant genes, including *CPT1B* and *CPT2*, decreased in an intrahepatic cholestasis model. They could partly be counteracted by pretreatment with a PPARα agonist, fenofibrate, which also conferred protection against the cholestatic liver injury (Zhao et al., 2017). In addition, a direct and specific increase of CPT1A in HepG2 cells plays a crucial role in the lipid-lowering and anti-inflammatory effects exerted by Ginkgo biloba extract, quercetin, kaempferol, and isorhamnetin (Wei et al., 2014).

#### 3.2.1 Nonalcoholic Fatty Liver Disease

As a common nexus of a metabolic and hepatic disease, NAFLD is a clinical syndrome that involves lesions in the hepatic lobule, hepatic steatosis, and fat piling pathological features, despite no history of excessive alcohol consumption (Dludla et al., 2020; Ni et al., 2020). Insulin resistance and impaired adipose tissue function are instrumental in promoting hepatic lipid accumulation with metabolic syndrome. In fact, enhanced lipid accumulation, abnormal inflammatory response, and oxidative stress underpinned the development, severity, and the progression of NAFLD (Dludla et al., 2020). Hepatic mitochondrial dysfunction is commonly found in patients with nonalcoholic steatohepatitis. Dysfunction of hepatic mitochondria, altered expression of genes associated with lipid metabolism, and changes in triglycerides, cholesterol, and acyl-carnitines were observed in mice, indicating an impaired mitochondrial carnitine shuttle (Schröder et al., 2016). Using either a Western-style diet or a methionine- and choline-deficient diet, mice with mitochondrial dysfunction developed severe steatohepatitis, which is characterized by lipid accumulation, immune cell infiltration, and hepatocyte ballooning (Schröder et al., 2016).

Since the establishment of the importance of lipid homeostasis and mitochondrial function in NAFLD, much effort has been made to develop therapeutic agents that target the process. One study showed that leucine-rich repeat kinase 2 (LRRK2) participates in the regulation of FAO, and its deficiency might promote inflammation in a palmitic acid-induced NAFLD mouse model. Furthermore, CPT1A, the critical enzyme of FAO, is positively modulated by LRRK2 via the activation of AMP-activated protein kinase (AMPK) and PPARα (Lin et al., 2020). In another study, Liou et al. (2018) reported that fisetin, a naturally abundant flavonoid isolated from various vegetables and fruits, could alleviate hepatic lipid metabolism and improve NAFLD in mice via the activation of the FAO pathway. Recently, Hwangbo et al. (2020) showed that auranofin might have potential as a candidate for improving NAFLD symptoms. Auranofin significantly suppressed lipid peroxidation, inflammatory activity, and hepatic steatosis of liver tissues in NAFLD mice induced by a high-fat diet, which attributed to the decreased expression of NADPH oxidase 4 and PPARγ. Therefore, we hypothesize that regulating mitochondrial FAO and maintaining lipid homeostasis may alleviate NAFLD.

#### 3.2.2 Hepatic Ischemia–Reperfusion Injury

Hepatic IR injury is a severe clinical issue that could lead to poor outcomes; furthermore, no effective therapies have been established (Wang W. et al., 2020; Ibrahim et al., 2021). The paradigm of hepatic IR follows two apparently separate phases, namely, the ischemic and reperfusion phases. The ischemic phase induces cellular metabolic disturbance due to glycogen consumption, ATP depletion, and lack of oxygen supply, whereas the reperfusion phase results in metabolic disturbance and an unusual immune-inflammatory response that involves both direct and indirect cytotoxic mechanisms (Zhai et al., 2013). Zhang et al. (2018) showed that the pathophysiology of hepatic IR injury is primarily marked by lipid metabolic reprogramming, which results in a secondary effect of inflammation, thereby highlighting the role of lipid metabolism in disease pathogenesis.

In view of the present research, FA metabolism has been attracting considerable interest in hepatic IR injury (Hwangbo et al., 2020), and the role of FAO in the disease is an important research topic (Wang et al., 2020). Luo et al. (2021) reported that CPT1A deficiency mitigated inflammation and oxidative stress in carbon tetrachloride-induced liver injury of mice. They also showed that CPT1A overexpression suppressed the nuclear factor erythroid-2-related factor 2/heme oxygenase-1 and nucleotide-binding oligomerization domain-like receptor protein 3 inflammasome signaling pathways. In a classic IR model with the mice exposed to *in situ* ischemia for 1 h and reperfusion for 6 h, Wang et al. (2020) found that mild hypothermia effectively attenuated hepatic IR injury, which might be attributable to the preservation of mitochondrial FAO and CPT1A expression via the Janus kinase 2/signal transducer and the activator of transcription 3 signaling. In addition, pharmacological interventions of FAO had obvious effects on IR injury, i.e., the activation of CPT1-dependent FAO by leptin significantly attenuated IR-induced injury, which is manifested by reduced hepatic enzyme level, hepatic injury score, hepatocyte apoptosis, and mitochondrial damage, while the inhibition of CPT1 by etomoxir imparted negative effects.

As an essential metabolic and digestive organ, the effect of the CPT system on hepatic disorders is comparatively definite. CPT deficiency and FAO downregulation induce lipid metabolic reprogramming, which leads to a secondary effect on inflammation in diseases. Thus, therapeutic manipulation targeting CPT system to maintain lipid homeostasis may be of great significance to treat multiple liver disorders.

### 3.3 Kidney Diseases

Kidneys are organs associated with high energy consumption, and they generate large amounts of ATP via FAO. The strongest mitochondrial FAO activity has been observed in the proximal and distal convoluted tubules (Wirthensohn and Guder, 1986). Tubular EpiCs have been confirmed to primarily rely on FAO as their energy source, whereas elevated CPT and FAO aggravated mitochondrial ROS accumulation and cell injury in diabetic nephropathy (Rosca et al., 2012). In human and murine models, reduced FAO contributed to the pathophysiology of kidney fibrosis (Kang et al., 2015). In addition, during the development of AKI, damage to the proximal tubule and medullary thick ascending limb resulted in reduced PPARα expression, which subsequently led to the diminished expression and activity of mitochondrial FAO enzymes, represented by the CPT system.

#### 3.3.1 Kidney Fibrosis

Kidney fibrosis is the major pathological process and common end point of the progression of chronic kidney disease, which eventually leads to end-stage renal disease (Zeisberg and Neilson, 2010). In addition to proper blood pressure and glycemic control, therapeutic options to deter or revert the development of fibrosis are quite limited. In recent years, studies have focused on the metabolic disturbances coexisting with renal fibrosis. Among these, FAO reduction became critical for energy failure in the tubulointerstitial compartment, thus leading to inflammatory cell infiltration and tissue fibrosis (Kang et al., 2015; Chung et al., 2019). In the unilateral ureteral obstruction and transforming growth factor (TGF)-β1–induced kidney fibrosis models, the defective FAO and decreased CPT1A expression occurred during the progression of the disease. C1q/tumor necrosis factor (TNF)–related protein 6 (CTRP6) is a recently identified adiponectin analog, and it has been downregulated in an animal model of kidney fibrosis. The use of human CTRP6 peptide could inhibit extracellular matrix deposition and promote FAO by upregulating CPT1A (Xie et al., 2021). Concerning the critical role of CPT1A in FAO, one study treated tubular EpiCs with the CPT1 inhibitor etomoxir, and the upregulated expression of genes related to fibrosis such as *α-smooth muscle actin* and *vimentin* was observed (Kang et al., 2015). Conversely, a synthetic CPT1 activator, C75, significantly reduced the symptom of kidney fibrosis in an FA-induced murine model (Kang et al., 2015). In addition, Verónica et al. constructed a conditional transgenic mouse model with CPT1A overexpression in tubular EpiCs that was subjected to three models of renal fibrosis. The mice exhibited reduced fibrotic markers expression, attenuated proinflammatory response, and alleviated EpiC damage, which might be mediated by restoring mitochondrial homeostasis (Miguel et al., 2021).

#### 3.3.2 Acute Kidney Injury

Lipid accumulation is related to various kinds of AKI or ischemic renal injury (IRI); however, its underlying causative factors and pathways remain unclear (Scantlebery et al., 2021). Indeed, following the onset of ischemia, the accumulation of cholesterol and triglycerides was apparently protective due to the buffer effect against FA; however, excess lipids during the progression of ischemia, displayed as droplets, could cause renal injury (Erpicum et al., 2018). In an IRI model, Scantlebery et al. (2021) identified a significant accumulation of cholesterol, specific phospholipids, and sphingolipids in the kidneys. Meanwhile, *in silico* analysis revealed that several energy and lipid metabolism pathways, including mitochondrial FAO, were downregulated 24 h after IRI, which could contribute to lipid accumulation. Furthermore, the decrease in CPT1 activity during renal IRI has also been observed, and this led to a reduced FA uptake and defective mitochondrial FAO. Idrovo et al. (2012) subjected rats to renal IRI by bilateral renal pedicle clamping with microvascular clips for 60 min, followed by administration of CPT1 agonist, C75, or vehicle, and they found that C75 recovered FAO, improved renal function, and attenuated tissue injury in the animal model. Beside IRI, infection is another important etiological factor for AKI. Both metabolic and inflammatory complications have been observed during sepsis or endotoxemia; however, the molecular mechanism responsible for these LPS-modulated metabolic changes remains elusive. In a murine sepsis model, LPS has been shown to suppress FAO by inhibiting the expression of associated genes, including *CPT1*, in kidney and liver tissues. This mechanism might rely on interleukin-1 receptor–associated kinase 1, which is one of the key Toll-like receptor (TLR) 4 intracellular signaling kinases (Maitra et al., 2009).

Existing studies have shown that mitochondrial dysfunction is observed in various nephropathies. Besides, the repairment of damaged renal cells largely depends on the ability of the mitochondria to restore ATP production. Accordingly, the preservation of CPT and FAO may attenuate or reverse renal failure, thus becoming a promising therapeutic target for the kidney diseases.

### 3.4 Cardiovascular Disorders

FAO serves a pivotal role in myocardial fuel selection, which is a key feature of the function and health of the heart. Recent studies have revealed that abnormal CPT expression or activity and impaired FAO could also contribute to the pathogenesis of multiple cardiovascular disorders.

#### 3.4.1 Cardiac Dysfunction

Energy metabolism suppression is one of the cornerstones of cardiac dysfunction in sepsis/endotoxemia. Systemic inflammatory responses, as well as superoxide, nitric oxide, and peroxynitrite could impair cardiac CPT1 activity *in vivo* and *in vitro*, thus leading to myocardial dysfunction (Eaton et al., 2003). In an LPS-induced rat model of neonatal sepsis, CPT1 activity was significantly decreased in the heart compared to other organs (Fukumoto et al., 2002). Similarly, in another experimental model of murine endotoxemia, excess inflammation markedly reduced cardiac FAO and mechanical function. In addition, aminocarnitine, a CPT2 specific inhibitor, resulted in the accumulation of FAO intermediates in the heart, which further exacerbated inflammatory cardiac dysfunction. By contrast, the activation of CPT1 by C75 could restore both cardiac and mitochondrial FAO without any effects on inflammatory gene expression or cardiac function. The results indicated that impaired CPT-dependent FAO was detrimental to cardiac injury during endotoxemia, but CPT/FAO restoration alone was not sufficient to recover cardiac function (Makrecka-Kuka et al., 2020).

Cardiovascular disease is considered as one of the main causes of mortality for diabetic patients (Stamler et al., 1993). Compared to nondiabetic patients, myocardial dysfunction incidence was much higher in patients with diabetes, which was due to cardiac metabolic disturbance characterized by high FFA and reduced glucose utilization (Herlitz et al., 1988). In an animal model, diabetic rats had higher body weight, larger left ventricular end-diastolic diameter, and longer QT interval, along with increased proinflammatory cytokines and CPT1 expression in the heart than healthy rats. Nevertheless, treatment with 1,25-dihydroxyvitamin D3 dramatically ameliorated cardiac function, inflammatory response, and CPT1-mediated FA metabolism in diabetic hearts (Lee et al., 2014). TNFα is an adipose-derived proinflammatory cytokine that induces myocardial contractile dysfunction of the cardiomyocytes. In TNFα-stimulated mouse cardiac muscle cells, sodium hydrosulfide ameliorated the impaired mitochondrial respiration and ATP production/synthesis, and attenuated excess oxidative stress, which might be due to the enhanced expression of metabolic indices such as CPT1. The study indicated the therapeutic potential of sodium hydrosulfide for inflammation-associated cardiac dysfunctions (Lee et al., 2019).

#### 3.4.2 Endothelial Dysfunction

Most ECs in a healthy person are quiescent, and they maintain barrier function and vasoregulation, and counteract thrombosis and vascular inflammation. EC metabolism has emerged as a novel and promising therapeutic target to block vascular dysregulation associated with various diseases. Glycolysis and FAO are key regulators of EC metabolism, which further influences their function and behavior (Draoui et al., 2017). ROS overproduction in EC plays a critical role in endothelial dysfunction, whereas mitochondrial ROS (mtROS) is essential to the pathogenesis of diabetic vascular complications. In bovine aortic ECs, Kajihara et al. (2017) found that during hypoglycemia, the activation of FAO followed by mtROS generation and vascular cell adhesion molecule-1 expression could induce endothelial dysfunction. Yet, these effects could be suppressed by treatment with the CPT inhibitor etomoxir. Using CPT1A-silenced ECs in the LPS model, Kalucka et al. (2018) demonstrated that the endothelial loss of FAO-controlling CPT1A promoted leukocyte infiltration and barrier disruption by elevating endothelial oxidative stress. More importantly, the supplementation of acetate could counter ROS-mediated EC dysfunction in CPT1A-deficient mice, providing therapeutic opportunities in related disorders. As one of the stimulators for the CPT system, the PPAR family is also present in the endothelium. PPARα regulated lipid metabolism and inhibited inflammatory response in vascular ECs. In the skeletal muscle and heart, PPARα has been shown to increase the mitochondrial FFA uptake and subsequent FAO through the activation of CPT1 (Fruchart et al., 1999).

In healthy adults, blood vessels are lined with a single monolayer of quiescent ECs that remains in this state for years (Eelen et al., 2018). However, upon ischemia or inflammatory injury, quiescent ECs immediately switch to a proliferative/angiogenic state to achieve tissue homeostasis (Kalucka et al., 2018). Draoui et al. (2017) found that CPT1 and FAO-upregulated proliferation in ECs played an essential role in lymphangiogenesis by promoting DNA synthesis. Meanwhile, CPT1 inhibition in blood vessels has also been shown to have potential therapeutic benefits by blocking pathological angiogenesis. Endothelial FAO is also a critical regulator of endothelial-to-mesenchymal transition (EndoMT), which is a cellular process required for normal heart valve development and is often initiated by the TGF-β family of ligands. However, deregulated EndoMT is associated with a wide range of disorders. Xiong et al. (2018) constructed a conditional mouse model of endothelial CPT2 deletion, and they demonstrated that the disrupted FAO augmented the magnitude of embryonic EndoMT, leading to the thickening of cardiac valves and elevated permeability of multiple vascular beds in adult mice. Soon after that, Shimizu et al. (2020) concentrated on how the TGF-β downstream pathway modulated CPT2 expression. They discovered that the protein kinase R-like endoplasmic reticulum kinase signaling was demanded for cardiac valve formation via CPT2-dependent FAO and EndoMT. Taken together, the results implicated that endothelial CPT and FAO were critical to maintain EC fate, and the therapeutic manipulation targeting EC metabolism might offer the basis for treating various EndoMT-linked disorders.

For the cardiovascular system, enormous quantities of energy are required to maintain the metabolism and physiological function. Current studies implicate the correlation between lipid metabolism reprogramming and cardiovascular disorders and provide evidence that the mitochondrial CPT system is essential for normal cardiac and EC function. Further research is needed to confirm the findings and to develop new effective drugs targeting CPT.

### 3.5 Cancers

Currently, metabolic rewiring, which supports unrestricted proliferation and metastatic progression of cancer cells, is widely accepted to be an emerging hallmark of cancers (Petővári et al., 2020). As the pivotal energy source and fundamental cellular components in tumor cells, FA is also involved in lipid-dependent metabolic reprogramming. A growing number of studies have pointed out that FAO and CPT are the key regulatory mechanisms underlying the survival, growth, and drug resistance of cancer cells, placing CPT as an emerging target for cancer therapeutics.

#### 3.5.1 Experimental and Preclinical Studies

In the tumor microenvironment, adipocytes served as a metabolic regulator and an energy provider to promote the survival and growth of several cancer cells. One research group reported that adipocytes supplied FA for rapid tumor growth, suggesting a significant role for lipid metabolism in the treatment of cancers (Nieman et al., 2011). Similarly, Wen et al. (2017) isolated adipocytes from tumor tissues of colon cancer patients and found a transfer of FFA from the adipocytes to the cancer cells. Through the absorption of FA, colon cancer cells are resilient to nutrient deprivation conditions as these are capable of upregulating mitochondrial FAO. In addition to colon cancer, Nieman et al. (2011) arrived at a similar conclusion in ovarian cancer. Although studies have confirmed the transfer of FAs from adipocytes to cancer cells, its underlying molecular mechanism remains unclear. In colon cancer patients, abundant adipocytes were correlated with the presence of invasive tumor cells. Xiong et al. (2020) demonstrated that CPT1A is upregulated in colon cancer cells after exposure to adipocytes or FA. Furthermore, three-dimensional culture studies showed that CPT1A is upregulated in tumor cells within adipose tissues compared to that not in direct contact with adipocytes, whereas CPT1A silencing reduces tumor organoid formation and downregulates genes associated with cancer stem cells. In addition, CPT1A-dependent FAO might be a key metabolic pathway that associates adipocytes to colon cancer cells.

Immune cell metabolism in the tumor microenvironment is also important to antitumor immune responses. Macrophages enhance the immunity by phagocyting and killing tumor cells. However, the specific mechanisms have been poorly understood. CpG oligonucleotide, a TLR9 agonist, enhanced the antitumor potential of macrophages by increasing FAO and shunting of acetyl-CoA toward lipid substances synthesis, which needed the involvement of CPT1A and ATP citrate lyase (Liu et al., 2019). Chronic inflammation was considered to participate in the occurrence and development of colon cancer. Arctigenin, the major active constituent of *Fructus arctii*, has been reported to alleviate colitis and protect against colon carcinogenesis in mice models. Mechanistically, Arctigenin downregulated CPT1-mediated FAO, which further inhibited nucleotide-binding oligomerization domain-like receptor protein 3 inflammasome assemblies in macrophages (Qiao et al., 2020). During the development of NAFLD-promoted hepatocellular carcinoma, intrahepatic CD4^+^ T cells are crucial for antitumor surveillance. In the lipid-rich liver environment, elevated CPT expression and FAO might increase mitochondrial ROS and lead to cell death of CD4^+^ T cells, thus promoting tumor formation in a murine model, which could be blocked by the CPT inhibitor perhexiline (Brown et al., 2018). Latest evidence has shown a correlation between the toleration of local dendritic cell (DC) in the tumor microenvironment and immune evasion. Zhao et al. (2018) reported a site of immune privilege established by melanomas that drove FAO in DCs via elevating the expression of CPT1A. This FAO shift increased the activity of immunosuppressive enzymes and promoted the generation of regulatory T cells, leading to tumor-mediated immune tolerance. These findings implicate a role for the metabolic reprogramming of local immune cells in the antitumor therapy.

Except for the effects of FA from adipocytes and immune cells, FAO alterations in tumor cells themselves also affect their proliferation and migration. Xu et al. (2020) showed that a high-dose dexamethasone-inhibited tumor progression was associated with the downregulation of FAO genes, including *CPT1A*. It could be decreased by the uptake and consumption of lipids and glucose in cancer cells, thus indicating the orchestration of microenvironmental inherent metabolic pathways related to FAO. Wang et al. (2020) prepared betulinic acid–loaded nanoliposomes and evaluated their anticancer effects on colorectal cancer cell lines. This nanodrug significantly suppressed cell proliferation via modulating potential FA metabolism targets and pathways, such as the CPT system, which might be an effective therapy adjuvant in colorectal cancer. In addition, CPT1 and CPT2 are highly expressed in chronic lymphoblastic leukemia cells (Gugiatti et al., 2018), while the inhibition of CPT by perhexiline led to a decreased FA transport, damaged mitochondrial integrity, and the death of leukemic cells (Liu et al., 2016). Tumor growth is an angiogenesis-dependent process that requires continuous neovascularization. Wang et al. (2019) demonstrated that interleukin-17 promoted tumor growth of human lung cancer cells *in vivo* and *in vitro*, as well as stimulated angiogenesis by enhancing FAO and the mitochondrial respiration of ECs, which could be blocked by using the CPT inhibitor etomoxir. Furthermore, CPT1A knockdown in breast cancer cells disrupts invasion and lymphangiogenesis of human dermal lymphatic ECs (HDLECs). In addition, CPT1A-null HDLECs showed compromised invasion and lymphangiogenesis relative to the negative control (Xiong Y. et al., 2018).

#### 3.5.2 Clinical Studies

Based on the above information, the CPT system becomes a potential target for the diagnosis and treatment of various cancers, which draws the attention of researchers and clinicians. Tan et al. (2021) collected serum from breast cancer patients, patients with benign breast disease, and healthy controls to estimate the accuracy of CPT1A as a marker in the diagnosis of breast cancer. The results showed that CPT1A levels were higher in patients than in controls, and they were dramatically associated with TNM stage, histological grading, and metastasis. This study suggested a remarkably high diagnostic efficiency of CPT1A that could serve as an indicator for breast cancer monitoring. Malignant melanoma pertains to an aggressive skin tumor with poor prognosis, with approximately 50% of patients harboring gain-of-function mutations in the *BRAF* gene. Treatment of *BRAF* ^V600E^-mutant melanomas using mitogen-activated protein kinase inhibitors (MAPKi) induces tumor regression; however, this eventually results in drug resistance. Aloia et al. (2019) analyzed freshly isolated tumor biopsies from metastatic stage IV melanoma patients with paired pretreatment and early-on treatment and found that melanoma cells treated with MAPKi exhibited increased levels of PPARα-mediated and CPT1A-dependent FAO. In addition, the concomitant inhibition of FAO and glycolysis could induce apoptosis in MAPKi-treated melanoma cells, possibly benefitting patients receiving MAPKi therapies. Research on gastric cancer also proved the relationship between the CPT system and the tumor growth–promoting effect. Chen et al. (2020) showed that hypoxia-induced high expression of CPT1C was closely associated with the poor prognosis of patients with gastric cancer and could promote the proliferation of gastric cancer cells, while gene silencing or etomoxir treatment significantly suppressed cell proliferation and caused cell cycle arrest. In addition, studies on colorectal cancer indicated the possibility of regarding FAO inhibition as a novel approach and clinical strategy against the disease. Through experiments in tissue samples of colorectal patients and human colorectal cancer cell lines, Wang et al. (2018) demonstrated that CPT1A-mediated FAO activation induces cancer cells to resist anoikis, which is a specialized form of apoptosis triggered by the loss of adhesion to the extracellular matrix.

The majority of cancer-related deaths have been attributed to highly aggressive metastases. Colorectal patients with peritoneal metastases have been associated with decreased overall survival. Peng et al. (2021) investigated the primary tumor tissues collected from patients with T4Nx colorectal cancer, and they determined that CPT1A was downregulated in patients with peritoneal metastases. Furthermore, cancer-associated fibroblasts (CAFs) promoted the proliferation, invasion, and migration of colon cancer cells by increasing CPT1A expression. Conversely, disrupting FAO in CAFs with the CPT inhibitor etomoxir results in a decrease in tumor growth and intraperitoneal dissemination. Mao et al. (2021) reported CPT1A expression in 325 cytogenetically normal acute myeloid leukemia (AML) patients, except those with solid tumors. The results revealed that AML patients with upregulated CPT1A expression have a relatively short overall survival than those with downregulated expression. CPT1A-selective inhibitor ST1326 in combination with B-cell lymphoma/leukemia-2 inhibitor ABT199 imparted strong synergistic inhibitory effects on AML cells as well as primary patient blasts.

The development of cancer is a complex pathological process involving multiple cells and mediators. The interaction between cells and microenvironment may induce metabolic reprogramming, thus affecting the prognosis. For the contradictory findings from Peng et al., we speculate that the energy provided by CAFs is sufficient to support distant metastasis of the tumors or there exists competition between primary tumor cells and CAFs for FA utilization. Current studies highlight the importance of lipid metabolic state of single cell type for the proliferation and migration of tumor cells. Novel techniques, such as single-cell sequencing, could be applied to further clarify the metabolic shift in each cell types and patterns of cell–cell cross talk in different cancers.

## 4 Conclusion and Perspectives

Lipids are important metabolic energy reserves and crucial components of cellular signal transduction pathways. Dysregulated lipid metabolism is involved in various diseases, including acute and chronic inflammatory disorders and cancers. As one of the most important steps of lipid metabolism, FAO and its key rate-limiting enzyme, the CPT system, regulate host immune responses, which is of great clinical significance. The deficiency or overactivation of the CPT system can ultimately lead to the disruption of immune homeostasis, and therefore elevate the risk for various inflammatory diseases and even cancers. Evidence has shown the involvement of the CPT system and related mitochondrial FAO in the development and progression of these disorders. Accordingly, agonists or inhibitors targeting the CPT system have emerged as novel therapies for these diseases. In addition to experimental therapeutic strategies, there have been several clinical trials on CPT modulators. Oral or intravenous administration of L-carnitine could significantly improve the condition of patients with chronic kidney disease (Nemati et al., 2019), septic shock (Puskarich et al., 2015; Evans et al., 2019), knee osteoarthritis (Malek et al., 2016), coronary artery disease (Lee et al., 2015), and maintenance hemodialysis (Savica et al., 2005). However, the efficacy, stability, and safety of the agents, as well as whether they could disturb local or systemic energy metabolism should be considered comprehensively in future clinical uses. Besides, due to the uncertain role of the CPT system in several diseases, such as COPD and cancers, new techniques should be applied to seek a breakthrough. For example, single-cell sequencing could be used to identify the expression and influence of the CPT system within each cell type and CRISPR-CAS9 gene editing could be utilized to modify CPT expression in specific cells, thus providing more precise tools to explore its cell-specific role. Furthermore, given the fact that danger signals often change multiple times in lipid metabolism, developing more potent activators/inhibitors that target multiple FAO signaling pathways, including the CPT system or combined with other anti-inflammatory/antitumor therapeutics, may facilitate translation of this promising strategy into clinical application.
